# Neutrophil-Lymphocyte Ratio as a Screening Test for Preeclampsia in the First and Early-Third Trimesters of Pregnancy: A Cohort Study: Neutrophil-Lymphocyte Ratio and Preeclampsia

**DOI:** 10.31661/gmj.v11i.2433

**Published:** 2022-12-26

**Authors:** Azam Faraji, Zahra Nourani, Elham Askary, Marjan Zare, Maryam Kasraeian

**Affiliations:** ^1^ Maternal-fetal medicine Research Center, Department of Obstetrics and Gynecology, School of Medicine, Shiraz University of Medical Sciences, Shiraz, Iran; ^2^ Department of Obstetrics and Gynecology, School of Medicine, Infertility Research Center, Shiraz University of Medical Sciences, Shiraz, Iran; ^3^ Maternal-fetal Medicine Research Center, Shiraz University of Medical Sciences, Shiraz, Iran

**Keywords:** Neutrophils, Lymphocytes, Preeclampsia, Gestation, Hypertension

## Abstract

**Background:**

Preeclampsia (PEC), affecting 5-10% of all pregnancies, is a multisystem disease diagnosed by hypertension (HTN) and protein in the urine or multi-organ problems without signs of proteinuria occurring after 20 weeks of gestation. PEC is the most common cause of maternal death and can lead to premature delivery. The current study aimed to investigate the accuracy of neutrophil-lymphocyte ratio (NLR) for predicting PEC in the first and early-third trimesters of pregnancy in a normal population in the south of Iran.

**Materials and Methods:**

A large-scale cohort study was performed from early pregnancy onward during November 2018-2019 in a normal population from Fars province, Southwest Iran. Four hundred forty-nine pregnant women were followed prospectively, and normal blood pressure (normotensive), gestational HTN, and PEC groups were compared in terms of white blood cells (WBC) count, neutrophil, lymphocyte, and NLR.

**Results:**

The serum levels of WBC count, neutrophil, and NLR significantly increased from the first to the early-third trimesters of pregnancy (P0.05); however, lymphocytes decreased (P0.05). The NLR cut-off points for predicting PEC were 2.79 (sensitivity=86.7% and specificity=92.6%) in the first and 3.2 (sensitivity=90.5% and specificity=79.4%) in the early-third trimesters.

**Conclusion:**

Our findings revealed that NLR could be an accurate predictive factor for PEC in the first and early-third trimesters of pregnancies; however, more studies are needed to investigate the immunomodulatory drugs for the prevention of PEC.

## Introduction

Preeclampsia (PEC) is a multisystem disease diagnosed by hypertension (HTN) and proteinuria or multi-organ problems without signs of proteinuria occurring after 20 weeks of gestation [1]. PEC affects approximately 5 to 10% of all pregnancies [2]. It is one of the three main causes of mortality and morbidity during pregnancy besides infection and bleeding [3]. Also, PEC is the most common cause of maternal death and can lead to premature delivery [2]. Hence, PEC has been considered an economic burden on the health system [4].

Although the definitive mechanism of PEC is not clear, two theories are considered. Abnormal placentation and vascular endothelial damage by oxidative stress cause endothelial dysfunction resulting in clinical manifestations of PEC [5]. Another mechanism of PEC is genetic and immunological factors [6][7][8]. Among the cited factors, maternal immunologic intolerance is among the full investigations.

Successful semi-allograft reaction in normal pregnancy and balance between the immune system and inflammatory responses are crucial for maintaining the pregnancy. Normal pregnancy and PEC are infl ammatory states of the body in which normal pregnancy controls the infl ammatory response, but PEC has an excessive systemic infl ammatory response (SIR) [9].

Lipids secreted from the placenta cause activation in the leukocyte classes in the intervillous space. The leukocyte re-enters the maternal systemic circulation, resulting in vascular dysfunction in women with PEC [10]. Some hematological parameters derived from the peripheral blood cells are known as SIR markers, including the neutrophil-lymphocyte ratio (NLR), platelet-lymphocyte ratio (PLR), red cell distribution width (RDW), and mean platelet volume (MPV) [10]. To lower adverse pregnancy outcomes, timely diagnosis of high-risk women prone to developing PEC is crucial. Nowadays, the healthcare system focuses on the prevention of PEC through modifications in lifestyle, nutritional supplementation, and pharmacological therapy [11].

In the second trimester of pregnancy, the maternal serum level of angiogenic and antiangiogenic factors besides abnormal uterine artery Doppler velocimetry provide a moderate to high predictive value to determine the early-onset PEC [12]. However, a low-cost screening test that is available and easy to use is necessary.

The complete blood count (CBC), including the neutrophil, lymphocyte, and NLR could have a diagnostic value for predicting PEC [13]. Most studies have reported significant predictive values of NLR for PEC in non-prospective designs [13][14][15][16][17][18][19]. Though studies investigated the diagnostic value of NLR in predicting PEC, further large-scale prospective studies are required to validate its accuracy [20][21]. Since the neutrophil, lymphocyte, and NLR are relatively inexpensive and available tests compared to other predictive tests, timely prediction, diagnosis, and management of PEC are important to lower adverse pregnancy outcomes. Hence, in a large-scale prospective cohort study, this study aimed to evaluate the predictive value of NLR for the detection of the PEC in the first and early-third trimesters of pregnancy.

## Materials and Methods

### Study Population and Design

A cohort study was performed on 449 healthy pregnant women from early pregnancy onward. All participants were recruited from dual centers affiliated with Shiraz University of Medical Science (SUMS) as the referral center in the south of Iran from November 2018 to November 2019.

The inclusion criteria were singleton pregnancy up to 30 weeks of gestational age with the live fetus, clear gestational age, and natural conception. The exclusion criteria were diabetes mellitus, heart failures, liver disorders, renal failures, chromosomal abnormalities, inherited metabolic diseases, thyroid disease, chronic hypertensive disorders, any disease that directly affects the WBC count (such as all rheumatologic diseases, vascular disorders, infections, and immunosuppression conditions), drug-induced immunosuppression, malignancies, and active allergic reaction [22].

All the participants were followed to the end of the pregnancy, and their blood pressures were checked during each visit. At the end of pregnancy, they were divided into two groups; patients with hypertensive pregnancy disorders (HPD), including gestational HTN and PEC, and patients without HTN (normotensive). Cases of PEC were considered severe if they had at least one of the following symptoms: a systolic blood pressure ≥160 mmHg, a diastolic blood pressure ≥110 mmHg, proteinuria of 5 g/24 hours, proteinuria of 3+ or more, oliguria, pulmonary edema, or convulsions/eclampsia. All other cases were considered mild [23].

### Sample Size Calculation

Considering α=0.05 a d the prevalence of PEC=0.05, we used the Buderer formula for diagnostic tests:


n \geq \frac{Z_{\alpha/2}^2 \cdot \text{sensitivity} \cdot (1 - \text{sensitivity})}{d^2 \cdot \text{prevalence}}

Where α = 0.05, Zₐ/₂ = 1.96

Using the information of NLR sensitivity equal to 73.4% from the previous article [24], a minimum of 400 participants were needed.

### CBC Analysis

K2-EDTA anticoagulated venous blood samples were taken after eight-hour overnight fasting from all the participants at the first (0-14 gestational weeks) and early-third trimesters (24-30 gestational weeks) of pregnancy to evaluate the levels of the WBC, neutrophil, lymphocyte, and NLR using the Sysmex XS-800i analyzer (Sysmex Corporation, Japan).

### Follow-up and Data Collections

All the participants completed a brief checklist about the maternal age, height, weight, parity, gravida, history of abortion, and gestational age at the time of entering the study. All women were followed-up during the whole pregnancy period. Also, one visit was done at weeks 6-12 postpartum to check the blood pressure. Any sign of HTN and PEC was evaluated precisely. Patients with rapid weight gain, edema, headache, blurred vision, epigastric pain, and unexplained nausea and/or vomiting were referred to the primatologists for further evaluation to detect any case of gestational HTN and PEC.

### Ethical Considerations

This study was approved by the Ethics Committee of SUMS (approval code: IR.SUMS.MED.REC.1397.446). Consent forms were taken from all participants, the process of the work was completely anonymous, and the results were reported to them.

### Statistical Analysis

Median±interquartile range (IQR=Q3-Q1) was used to describe variables quantitatively, and frequency (proportional frequency) was used to describe variables qualitatively. Kolmogorov-Smirnov normality, Chi-square, Fisher Exact, Mann-Whitney U, Wilcoxon rank, and Generalized Linear Models, as well as receiver operating characteristic (ROC) analysis with Uden index=sensitivity+specificity-1 (to determine cut-offs), were used. In addition, the positive predictive value (PPV) and negative predictive value (NPV) were calculated. All the analyses were done by SPSS Statistics for Windows (IBM SPSS Statistics for Windows, Version 23.0. Armonk, NY, USA). The significance level was considered at P=0.05

## Results

**Table:** Table1. Maternal Features of Pregnant Women

**Variables**		**Total (n=449)**		**HPD**		**P-value**	**PEC groups**		**P-value**
			Normotensive (n=408)	Gestational HTN (n=11)	PEC (n=30)		Mild (n=21)	Severe (n=9)	
	16-24	78 (17.4)	73 (17.9)	0 (0)	5 (16.7)		4 (19.05)	1 (11.1)	
**Age, y**	25-34	269 (59.9)	243 (59.6)	7 (63.6)	19 (63.3)	0.9	13 (61.9)	6 (66.7)	0.86
	35	102 (22.7)	92 (22.5)	4 (36.4)	6 (20)		4 (19.05)	2 (22.2)	
	≤18.5	17 (3.8)	17 (4.2)	0 (0)	0 (0)		0 (0)	0 (0)	
**BMI**	18.5-24.9	93 (20.7)	85 (20.8)	0 (0)	8 (26.7)	0.86	4 (19)	4 (44.4)	0.3
	≥25	339 (75.5)	306 (75)	11 (100)	22 (73.3)		17 (81)	5 (55.6)	
**Parity**	Null	192 (42.8)	177 (43.4)	3 (27.3)	12 (40)	0.24	9 (42.9)	3 (33.3)	0.62
	Multi	257 (57.2)	231 (56.6)	8 (72.7)	18 (60)		12 (57.1)	6 (66.7)	

**HPD:** Hypertension pregnancy disorder; **BMI:** Body mass index; **PEC:** Preeclampsia ^*^Data presented as n (%)

**Table:** Table2. White Blood Cells, Neutrophil, Lymphocyte, and Neutrophil-Lymphocyte Ratios in the First and Early-Third Trimesters of Pregnancy among 449 Pregnant Women

Parameters				Groups (Median±IQR)	
		Normotensive	Gestational HTN	Mild PEC	Sever PEC
	WBC	7500±1900	7000±900	^*^8100±1250	7500±2010
First trimester	Neutrophil (%)	64±6	69±4	^*&^71±2	^*&^71±2
	Lymphocyte (%)	29±6	28±7	^*&^24±3	^*&^23±2
	NLR	2.21±0.62	2.3±0.91	^*&^2.96±0.4	^*&^3.13±0.25
	WBC	^†^9200±1500	^†^70±4	^*&†^9900±3150	^*†^9700±2600
Early-third trimester	Neutrophil (%)	^†^69±5	^†^24±6	^*&†^76±4	^*&†^75±2
	Lymphocyte (%)	^†^25±5	^†^3.12±0. 94	^*&†^20±4.5	^*&†^18±5
	NLR	^†^2.77±0.69	^†^70±4	^*&†^3.8±1.14	^*&†^4.1±1

**WBC:** White blood cells; **NLR:** Neutrophil-lymphocyte ratio; **HTN**: Hypertension; **PEC:** Preeclampsia; **IQR:** Inter quartile range ^*^ Significant difference vs. normotensive group ^&^ Significant difference vs. gestational HTN group ^†^ Significant difference vs. first trimester

**Table:** Table3. Associations between Various Groups of Pregnant Women in the Terms of White Blood Cells, Neutrophil, Lymphocyte, and Neutrophil-Lymphocyte Ratios in the First and Early-Third Trimesters of Pregnancy

Parameters		Normotensive vs.		Gestational HTN vs.	
		Mild PECOR (95% CI)	Severe PECOR (95% CI)	Mild PECOR (95% CI)	Severe PECOR (95% CI)
	WBC	1.001 (1-1.001)	1 (1-1)	1(1-1)	1(1-1)
First trimester	Neutrophil (%)	1.017 (1.013-1.021)	1.008 (1.005-1.01)	1.08(1.06-1.11)	1.07(1.04-1.1)
	Lymphocyte (%)	0.987 (0.983-0.992)	0.993 (0.99-0.998)	0.94(0.91-0.98)	0.93(0.89-0.98)
	NLR	1.18 (1.13-1.23)	1.09 (1.06-1.12)	1.69(1.33-2.15)	1.67(1.35-2.07)
	WBC	1.001 (1-1.001)	1.001 (1-1.003)	1(1-1.001)	1(1-1)
Early-third trimester	Neutrophil (%)	1.021 (1.01-1.025)	1.008(1.005-1.01)	1.07(1.04-1.09)	1.11(1.06-1.15)
	Lymphocyte (%)	0.985 (0.98-0.989)	0.992 (0.98-0.996)	0.95(0.92-0.98)	0.92(0.88-0.96)
	NLR	1.3 (1.11-1.17)	1.07 (1.04-1.09)	1.16(1.04-1.29)	1.67(1.34-2.07)

**WBC:** White blood cells; **NLR:** Neutrophil-lymphocyte ratio; **HTN**: Hypertension; **PEC:** Preeclampsia; **OR:** Odds ratio;**CI:** Confidence interval

**Table:** Table4. Cut-off Point Values of the Neutrophil Percent and Neutrophil-Lymphocyte Ratio for Prediction of Preeclampsia among Pregnant Women Based on the First and Early-Third Trimesters of Pregnancy

	Parameters		Cut-off point	AUR	Sensitivity	Specificity	PPV (%)	NPV (%)
		PEC	69.5	0.98	100	97.6	45.4	100
	Neutrophil (%)	Mild PEC	69.5	0.97	100	0.96	98.5	48.4
First trimester		Sever PEC	69.5	0.97	100	0.96	98.5	100
		PEC	2.79	0.93	86.7	92.6	49.2	89
	NLR	Mild PEC	2.79	0.92	85	90.9	96.5	38.8
		Sever PEC	2.79	0.95	85.7	90.9	96.5	67.9
		PEC	72.5	0.96	96.7	86.9	49.2	89
	Neutrophil (%)	Mild PEC	72.5	0.95	95.2	85	96.5	38.8
Early-third trimester		Sever PEC	72.5	0.93	95.2	85	96.5	97.5
		PEC	3.2	0.91	90.5	79.4	92.9	70.8
	NLR	Mild PEC	3.2	0.92	90.51	79.4	92.9	38.1
		Sever PEC	3.2	0.91	90.5	79.4	92.9	73.5

**PEC:** Preeclampsia; **NLR:** Neutrophil-lymphocyte ratio; **AUR:** Area under ROC; **PPV:** Positive predictive value; **NPV:** Negative predictive value

Maternal features of pregnant women are presented in Table-1. In this study, 449 pregnant women were followed prospectively; of them, 408 (90.9%), 11 (2.4%), and 30 (6.7%) women were normotensive, gestational HTN, and PEC, respectively. Also, 70% and 30% of the PEC women had mild and severe PEC, respectively. Age, body mass index (BMI), and parity were not significantly different among normotensive, gestational HTN, and PEC groups (P>0.05, Table-1). Also, there were no significant differences between mild and severe PEC groups regarding age, BMI, and parity (P>0.05, Table-1).

Regarding Table-2, the WBC count, neutrophil, and NLR of all groups significantly increased from the first to the early-third trimesters (P<0.001); however, the lymphocyte significantly decreased (P<0.001). Also, there were no significant differences in terms of WBC count, neutrophil, lymphocyte, and NLR between normotensive and gestational HTN groups in both the first and early-third trimesters (P>0.05).

As mentioned in Table-3, the neutrophil, WBC, and NLR were significantly higher in the mild PEC group compared to the normotensive group in the first and early-third trimesters (P<0.05); however, the lymphocyte was significantly lower in the first and early-third trimesters (P<0.05). Also, the strongest associations were for NLR in the first and early-third trimesters (odds ratio [OR]=1.18 and 1.3, respectively).

The neutrophil and NLR were significantly higher in the severe PEC group compared to the normotensive group in the first and early-third trimesters (P<0.05, Table-3); however, the lymphocyte was significantly lower (P<0.05); despite no significant difference in WBC count in the first trimester (P>0.05), it was significantly higher in the early-third trimester (P<0.05, Table-3). The strongest associations were for NLR in the first and early-third trimesters (OR=1.09 and 1.07, respectively).

The neutrophil and NLR were significantly higher in the mild PEC group compared to the gestational HTN group in both the first and early-third trimesters (P<0.05, Table-3); however, the lymphocyte was significantly lower (P<0.05). Although there was no association for WBC in the first trimester (P>0.05), it was significantly higher in the early-third trimester (P<0.05, Table-3); the strongest associations were for NLR in the first and early-third trimesters (OR=1.69 and 1.16, respectively).

The neutrophil and NLR were significantly higher in the severe PEC group compared to the gestational HTN group in the first and early-third trimesters (P<0.05, Table-3); however, the lymphocyte was significantly lower (P<0.05); also, WBC count did not differ in the first and early-third trimesters (P>0.05). The strongest associations were for NLR in the first and early-third trimesters (OR=1.67 and 1.67), respectively.

The cut-off points of the neutrophil and NLR in the first and early-third trimesters of pregnancy for PEC, mild PEC, and severe PEC are shown in Table 4. The neutrophil of more than 69.5% and 72.5% in the first and early-third trimesters could validly predict PEC, mild PEC, and severe PEC, respectively (Table-4). Also, the NLR of more than 2.79 and 3.2 could predict PEC, mild PEC, and severe PEC in the first and early-third trimesters, respectively (Table-4).

## Discussion

Totally, 449 pregnant women were followed prospectively, of whom 90.9%, 2.4%, and 6.7% had normal hypertension, gestational HTN, and PEC, respectively. Maternal age, BMI, and parity did not differ among the normotensive, PEC, and gestational HTN groups. The serum levels of the WBC count, neutrophil, and NLR increased significantly from the first to the early-third trimesters of pregnancy; however, the lymphocyte decreased. The WBC count, neutrophil, and NLR were significantly higher in the PEC group compared to the normotensive group in the first and early-third trimesters; however, the lymphocyte was lower. The NLR and neutrophil cut-off points for predicting PEC were 2.79 and 69.5% in the first, and 3.2 and 72.5% in the early-third trimesters, respectively.

NLR is proven as a marker of the SIR and is reported in some diseases, including appendicitis, advanced stages of cancer, acute myocardial infarction, acute pulmonary embolism, and ulcerative colitis [25][26][27][28].

Various studies have shown that WBC types, e.g., neutrophils, are associated with inflammatory responses, namely atherogenesis and atheroma thrombosis [29]. Maternal leukocytes are activated during pregnancy as well as PEC [29][30]. Kurt *et al*. reported higher neutrophils in patients with PEC, indicating an increased inflammatory status [31].

In PEC, hyper-activation of the inflammatory cell and immunological responses are the causes of releasing inflammatory cytokines, antibodies, and oxidative agents, resulting in endothelial dysfunction [15]. PEC is associated with impaired regulation of TH_1_and TH _2_ as inflammatory responses and consequently increased NLR [14][15][16][17][19].

Yavuzcan *et al*. reported that, though not statistically significant, NLR was higher in PEC women than normal women [32]; however, several authors have reported a statistically significant positive association, suggesting that NLR could predict PEC and its severity [33][34][35].

Kang *et al*. showed that the amount of NLR was higher in patients with PEC, especially in severe cases [15]. While Kurt *et al*. reported that NLR was not significantly different between PEC and normal groups, but the neutrophil was significantly higher in the group with PEC [31]. However, recently published articles have reported that NLR was significantly higher in PEC women [36][37]. Also, in the current study, the neutrophil showed a more accurate predictive value for PEC in both the first and early-third trimesters. In other words, the sensitivity and specificity of the neutrophil were more than those of NLR. In line with our study, Canzoneri *et al*. showed that neutrophils increased in PEC [38]. Also, they reported leukocytosis in PEC patients, which is associated with the severity and degree of thrombocytopenia in Hemolysis, Elevated Liver enzymes, and Low Platelets syndrome [38][39].

Although we performed this study with a large sample size and prospective design, an underestimation in reporting the prevalence of HPD due to excluding the high-risk women from the study should be considered as the main limitation of our study.

## Conclusion

Regarding routine CBC checking in the first trimester and 24-28 weeks of pregnancy, measured NLR and neutrophil in the first and early-third trimesters of pregnancy could be accurate predictions of PEC in a normal population.

## Conflict of Interest

The authors declared no competing interest in the study.

## References

[R1] The American (2013).

[R2] Foo L, Tay J, Lees CC, McEniery CM, Wilkinson IB (2015). Hypertension in pregnancy: natural history and treatment
options. Curr Hypertens Rep.

[R3] Main EK, McCain CL, Morton CH, Holtby S, Lawton ES (2015). Pregnancy-related mortality in California: causes,
characteristics, and improvement opportunities. Obstet Gynecol.

[R4] Fox A, McHugh S, Browne J, Kenny LC, Fitzgerald A, Khashan AS, et al (2017). Estimating the Cost of Preeclampsia in the Healthcare System:
Cross-Sectional Study Using Data From SCOPE Study (Screening for Pregnancy
End Points). Hypertension.

[R5] Steegers EA, Von Dadelszen, Duvekot JJ, Pijnenborg R (2010). Pre-eclampsia. The Lancet.

[R6] Boeldt DS, Bird IM (2017). Vascular adaptation in pregnancy and endothelial dysfunction in
preeclampsia. J Endocrinol.

[R7] Hansen AT, Bernth Jensen, Hvas AM, Christiansen M (2018). The genetic component of preeclampsia: A whole-exome sequencing
study. PLoS One.

[R8] Michita RT, Kaminski VL, Chies JAB (2018). Genetic Variants in Preeclampsia: Lessons From Studies in
Latin-American Populations. Front Physiol.

[R9] Stojanovska V, Zenclussen AC (2020). Innate and adaptive immune responses in HELLP syndrome. Front Immunol.

[R10] Walsh SW, Nugent WH, Archer KJ, Al Dulaimi, Washington SL, Strauss JF (2022). Epigenetic Regulation of Interleukin-17-Related Genes and Their
Potential Roles in Neutrophil Vascular Infiltration in Preeclampsia. Reprod Sci.

[R11] Poon LC, Sahota D (2019). Screening and prevention of preeclampsia. Matern Fetal Med.

[R12] Li L, Zheng Y, Zhu Y, Li J (2016). Serum biomarkers combined with uterine artery Doppler in
prediction of preeclampsia. Exp Ther Med.

[R13] Çintesun E, Incesu Çintesun, Ezveci H, Akyürek F, Çelik Ç (2018). Systemic inflammatory response markers in preeclampsia. J Lab Physicians.

[R14] Ghelfi AM, Lassus MN, Diodati S, Hails EA (2019). [Utility of the neutrophil/lymphocyte ratio and the
polymorphonuclear/ monomorphonuclear ratio, in the prediction of
preeclampsia]. Hipertens Riesgo Vasc.

[R15] Kang Q, Li W, Yu N, Fan L, Zhang Y, Sha M, et al (2020). Predictive role of neutrophil-to-lymphocyte ratio in
preeclampsia: A meta-analysis including 3982 patients. Pregnancy Hypertens.

[R16] Mannaerts D, Heyvaert S, De Cordt, Macken C, Loos C, Jacquemyn Y (2019). Are neutrophil/lymphocyte ratio (NLR), platelet/lymphocyte ratio
(PLR), and/or mean platelet volume (MPV) clinically useful as predictive
parameters for preeclampsia. J Matern Fetal Neonatal Med.

[R17] Örgül G, Aydın Haklı, Özten G, Fadiloğlu E, Tanacan A, Beksaç MS (2019). First trimester complete blood cell indices in early and late
onset preeclampsia. Turk J Obstet Gynecol.

[R18] Sitotaw C, Asrie F, Melku M (2018). Evaluation of platelet and white cell parameters among pregnant
women with Preeclampsia in Gondar, Northwest Ethiopia: A comparative
cross-sectional study. Pregnancy Hypertens.

[R19] Wang J, Zhu QW, Cheng XY, Liu JY, Zhang LL, Tao YM, et al (2019). Assessment efficacy of neutrophil-lymphocyte ratio and
monocyte-lymphocyte ratio in preeclampsia. J Reprod Immunol.

[R20] Hai L, Hu Z-D (2020). The clinical utility of neutrophil to lymphocyte ratio in
pregnancy related complications: a mini-review. J Lab Precis Med.

[R21] Mannaerts D, Heyvaert S, De Cordt, Macken C, Loos C, Jacquemyn Y (2019). Are neutrophil/lymphocyte ratio (NLR), platelet/lymphocyte ratio
(PLR), and/or mean platelet volume (MPV) clinically useful as predictive
parameters for preeclampsia. J Matern Fetal Neonatal Med.

[R22] Mayrink J, Souza RT, Feitosa FE, Rocha Filho, Leite DF, Vettorazzi J, et al (2019). Incidence and risk factors for Preeclampsia in a cohort of
healthy nulliparous pregnant women: a nested case-control study. Sci Rep.

[R23] Bodnar LM, Simhan HN, Catov JM, Roberts JM, Platt RW, Diesel JC, et al (2014). Maternal vitamin D status and the risk of mild and severe
preeclampsia. Epidemiology.

[R24] Panwar M, Kumari A, Hp A, Arora R, Singh V, Bansiwal R (2019). Raised neutrophil lymphocyte ratio and serum beta hCG level in
early second trimester of pregnancy as predictors for development and
severity of preeclampsia. Drug Discov Ther.

[R25] Avci B, Avci A, Dönmez Y, Kaya A, Gülen M, Özer A, et al (2020). The Effectiveness of Neutrophil-Lymphocyte Ratio in Predicting
in-Hospital Mortality in Non-ST-Elevation Myocardial Infarction. Emerg Med Int.

[R26] Hajibandeh S, Hajibandeh S, Hobbs N, Mansour M (2020). Neutrophil-to-lymphocyte ratio predicts acute appendicitis and
distinguishes between complicated and uncomplicated appendicitis: A
systematic review and meta-analysis. Am J Surg.

[R27] Phan T, Brailovsky Y, Fareed J, Hoppensteadt D, Iqbal O, Darki A (2020). Neutrophil-to-Lymphocyte and Platelet-to-Lymphocyte Ratios
Predict All-Cause Mortality in Acute Pulmonary Embolism. Clin Appl Thromb Hemost.

[R28] Yin X, Wu L, Yang H, Yang H (2019). Prognostic significance of neutrophil-lymphocyte ratio (NLR) in
patients with ovarian cancer: A systematic review and meta-analysis. Medicine (Baltimore).

[R29] Vecchié A, Bonaventura A, Carbone F, Maggi D, Ferraiolo A, Carloni B, et al (2018). C-reactive protein levels at the midpregnancy can predict
gestational complications. Biomed Res Int.

[R30] Gomes VJ, Nunes PR, Matias ML, Ribeiro VR, Devides AC, Bannwart-Castro CF, et al (2020). Silibinin induces in vitro M2-like phenotype polarization in
monocytes from preeclamptic women. Int Immunopharmacol.

[R31] Kurt RK, Aras Z, Silfeler DB, Kunt C, Islimye M, Kosar O (2015). Relationship of red cell distribution width with the presence and
severity of preeclampsia. Clin Appl Thromb Hemost.

[R32] Yavuzcan A, Caglar M, Ustun Y, Dilbaz S, Yidiz E, Ozbilgec S, et al (2014). Mean platelet volume, neutrophil-lymphocyte ratio and
platelet-lymphocyte ratio in severe preeclampsia. Ginekol Pol.

[R33] AKIL MA, Bilik MZ, Acet H, Tunç SY, Ertaş F, AYDIN M, et al (2015). Mean platelet volume and neutrophil lymphocyte ratio as new
markers of preeclampsia severity. Koşuyolu Heart Journal.

[R34] Kirbas A, Ersoy AO, Daglar K, Dikici T, Biberoglu EH, Kirbas O, et al (2015). Prediction of Preeclampsia by First Trimester Combined Test and
Simple Complete Blood Count Parameters. J Clin Diagn Res.

[R35] Serin S, Avcı F, Ercan O, Köstü B, Bakacak M, Kıran H (2016). Is neutrophil/lymphocyte ratio a useful marker to predict the
severity of pre-eclampsia. Pregnancy Hypertens.

[R36] Kurtoglu E, Kokcu A, Celik H, Tosun M, Malatyalioglu E (2015). May ratio of neutrophil to lymphocyte be useful in predicting the risk of developing preeclampsia? A pilot study. J Matern Fetal Neonatal Med.

[R37] Oylumlu M, Ozler A, Yildiz A, Oylumlu M, Acet H, Polat N, et al (2014). New inflammatory markers in pre-eclampsia: echocardiographic epicardial fat thickness and neutrophil to lymphocyte ratio. Clin Exp Hypertens.

[R38] Canzoneri BJ, Lewis DF, Groome L, Wang Y (2009). Increased neutrophil numbers account for leukocytosis in women
with preeclampsia. Am J Perinatol.

[R39] Terrone DA, Rinehart BK, May WL, Moore A, Magann EF, Martin Jr (2000). Leukocytosis is proportional to HELLP syndrome severity: evidence
for an inflammatory form of preeclampsia. South Med J.

